# Impact of intestinal microbiota on metabolic toxicity and potential detoxification of amygdalin

**DOI:** 10.3389/fmicb.2022.1030516

**Published:** 2022-11-24

**Authors:** Qiuyu Wen, Shen Yu, Shanshan Wang, Yan Qin, Quan Xia, Sheng Wang, Guanjun Chen, Chenlin Shen, Shuai Song

**Affiliations:** ^1^School of Pharmacy, The First Affiliated Hospital of Anhui Medical University, Anhui Medical University, Hefei, China; ^2^The Grade 3 Pharmaceutical Chemistry Laboratory of State Administration of Traditional Chinese Medicine, Hefei, China; ^3^Center for Scientific Research of Anhui Medical University, Hefei, China

**Keywords:** amygdalin, toxicokinetic, intestinal microbiota, cyanide, detoxification

## Abstract

Amygdalin (Amy) is metabolized into cyanide *in vivo*, which may lead to fatal poisoning after oral administration. The defense mechanisms against toxic cyanide have not yet been adequately studied. In this study, comparative toxicokinetics study of Amy was performed in normal and pseudo germ-free rats. The efficiency of cyanide release was significant higher in normal group when given a single oral dose of 440 mg/kg (50% median lethal dose). Thiocyanate, the detoxification metabolite, was firstly detected in feces, caecum, and intestinal microbiota incubation enzymic system. The results suggest intestinal microbiota is involved in bidirectional regulation of toxicity and detoxification of Amy. We further identified the species related to cyanogenesis of Amy with metagenomic sequencing, such as *Bifidobacterium pseudolongum*, *Marvinbryantia formatexigens*, and *Bacteroides fragilis*. Functional analysis of microbiota reveals the detoxification potential of intestinal microbiota for cyanide. Sulfurtransferase superfamily, such as rhodanese, considered as main detoxification enzymes for cyanide, are largely found in *Coriobacteriaceae bacterium*, *Butyricicoccus porcorum*, *Akkermansia muciniphila*, etc. Besides, cyanoamino acid metabolism pathway dominated by *Escherichia coli* may contribute to the detoxification metabolism of cyanide. In summary, intestinal microbiota may be the first line of defense against the toxicity induced by Amy.

## Introduction

Amygdalin (Amy), a typical representative of cyanogenic glycosides, is frequently found in the seeds of Rosaceae such as almonds and peach kernel (Bolarinwa et al., 2014). Amy, also known as vitamin B17, had been used to treat cancer in the 1980s, but its acute cyanide (CN) toxicity and poor curative effects for cancer caused controversy (Milazzo and Horneber, 2015). β-glucosidase is a key enzyme for CN release from Amy and the lower expression of rhodanese is observed in tumor tissue compared to the normal tissue (Ramasamy et al., 2006). Based on the cytotoxicity of CN, the combination of β-glucosidase and Amy targeting tumor tissue may provide a new viewpoint for Amy anti-tumor research (Zhou et al., 2012, 2020). Amy has been found effective in anti-inflammatory (Zhang et al., 2022a), immunomodulatory (Elsaed, 2019), anti-atherosclerosis (Wang et al., 2021), and antioxidation (Chen et al., 2021), however, there is no reliable evidence for the alleged curative effects of Amy itself on cancer patients.

Amy can be rapidly metabolized into prunasin by β-glucosidase after oral administration, and further hydrolyzed into hydrocyanic acid (Beamer et al., 1983; Shim and Kwon, 2010; Zhang et al., 2022b). Small amounts of CN will be converted into thiocyanate (SCN) by host detoxification enzymes involved in xenobiotic metabolism, especially in the mitochondria of the cell (Devlin et al., 1989; Wrobel et al., 2004). Excessive CN ion can be bound to Fe^3+^ in the Complex IV of the mitochondrial electron transport chain (also known as cytochrome c oxidase) and inactivate the enzyme, leading to interruption of intracellular respiration, blockage of electron transfer and oxidative phosphorylation, and inhibition of ATP synthesis (Yoshikawa et al., 1985; Downs et al., 2010). It is reported that the oral toxicity of Amy is significantly higher than that after intravenous administration due to the extensive metabolism by β-glucosidase expressed in intestinal microbiota (Moertel et al., 1981). As an additional metabolic “organ,” intestinal microbiota is involved in the metabolism of some drugs, such as digoxin (Haiser et al., 2013), L-dopa (Jameson and Hsiao, 2019), and irinotecan (Alexander et al., 2017). Previous studies showed that intestinal microbiota may affect the metabolism toxicity of Amy (Strugala et al., 1986), and probably participate in the detoxification of CN. Giant pandas, for example, do not experience CN poisoning even when they consume large amounts of bamboo shoots (Huang et al., 2016; Zhu et al., 2018).

**Figure 1:**
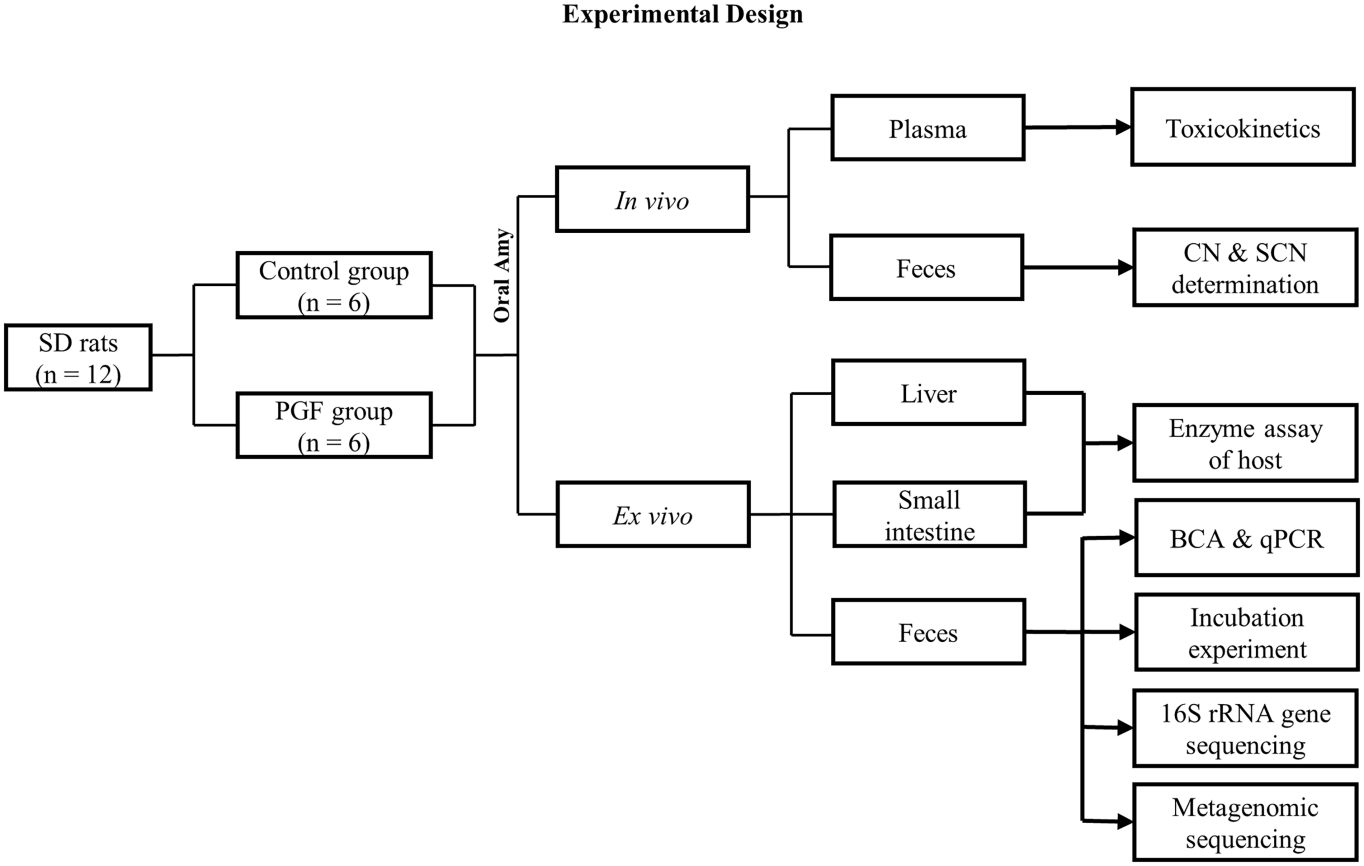
Flow diagram of the Experimental design.

In this study, we design a pseudo-germ-free (PGF) model, with a lower abundance of intestinal microbiota, to compare the differences in the kinetic processes of CN and SCN generation between the PGF and control groups *in vivo* and *ex vivo* (Figure 1). It is predicted that microbial detoxification of Amy might already occur prior to the metabolism of small intestine such that the rats experience limited absorption and systemic exposure to toxic compounds.

## Materials and methods

### Chemicals and materials

Amy, 2,3,4,5,6-pentafluorobenzyl bromide (PFB-Br), ethyl acetate, sodium tetraborate, and tetrabutylammonium sulfate (TBAS, 50% w/w solution in water) were purchased from Sigma-Aldrich Co. Ltd. (St. Louis, MO, United States). 1, 3, 5-tribromobenzene (TBB), used as internal standard (IS), was purchased from J&K Scientific Co. Ltd. (Beijing, China). Neomycin sulfate, ampicillin sodium, and metronidazole were purchased from Aladdin Co., Ltd. (Shanghai, China), and vancomycin hydrochloride was provided by Macklin Co., Ltd. (Shanghai, China). Ultra Taq PCR Mix (2×), G5 High-Fidelity DNA Polymerases, 2× S6 Universal SYBR qPCR Mix, and all primers were purchased from EnzyArtisan Co., Ltd. (Shanghai, China). TIANamp Stool DNA Kit was purchased from Tiangen Biotech Co., Ltd. (Beijing, China). BCA Protein Assay Kit was purchased from Beyotime Biotechnology Co., Ltd. (Shanghai, China). β-glucosidase Assay Kit was purchased from Solarbio Science & Technology Co.,Ltd. (Beijing, China). Rat Rhodanese ELISA Kit was purchased from MEIMIAN Industry Co., Ltd. (Jiangsu, China).

### Animals

Specific pathogen-free (SPF) male Sprague–Dawley (SD) rats (250 ± 20 g) were purchased from the Laboratory Animal Center of Anhui Medical University with certificate number SCXK 2017-001. All animal experiments and the related operating methods were conducted in compliance with the Experimental Animal Ethics Committee of Anhui Medical University (LLSC20170348). All animals were raised in SPF environment, the temperature was kept at 22–25°C, and the light and darkness alternated 12/12 h. Drinking water and feed were autoclaved, provided by the Experimental Animal Center of Anhui Medical University, and met the SPF standard.

### Establishment and evaluation of PGF model

All rats were housed in the laboratory for 1 week for acclimatization before the experiment and randomized into a control group (*n* = 6) and a PGF group (*n* = 6). An antibiotic cocktail consisting of 1 g/l neomycin sulfate, 1 g/l metronidazole, 1 g/l ampicillin sodium, and 0.5 g/l vancomycin hydrochloride in autoclaved water, was given to the PGF group for the suppression of intestinal microbiota for a week based on our preliminary exploration (Seki et al., 2007). Simultaneously, the control group was given the same drinking water, but the antibiotics were removed. All animals had free access to water and feed. The feed intake, water intake, and body weight were recorded during the period. Freshly voided fecal samples were collected on the seventh day for the following experiment.

To evaluate the PGF model, the total protein concentration and total DNA abundance were examined using the above fecal samples from the control and PGF groups, respectively.

The total protein content of intestinal microbiota from the PGF and control groups was detected with the bicinchoninic acid (BCA) method (*n* = 6). The total protein was extracted from feces according to a modified version of a previously published method (Kim et al., 2016). Approximately 0.5 g fecal sample we collected above were diluted in a 10 ml ice-cold PBS buffer and vortexed to mix thoroughly. The mixture was centrifuged at 3000 ×*g* for 15 min at 4°C to remove debris and impurities. The supernatant was discarded and another 10 ml ice-cold PBS was added to suspend the microorganism into the buffer, centrifuging at 300 ×*g* for 5 min at 4°C and the supernatant was collected, repeating 3 times. The gathered microbial suspension was centrifuged at 14000 ×*g* for 30 min at 4°C to separate the microbiota. A 2 ml ice-cold PBS was added to the sediment and the mixture was ground in a high throughput tissue homogenizer. The homogenate was further sonicated (300 W, work for 5 s /pause for 5 s) for 16.5 min in an ice bath. And the supernatant was finally separated by centrifugation at 18407 ×*g* for 30 min at 4°C for the BCA assay and incubation experiment. The BCA method was conducted according to the manufacturer’s introduction.

A qPCR experiment was conducted to quantify the relative abundances of the 16S rRNA gene in the feces of two groups (*n* = 6). The total DNA of microbiota extracted from 200 mg of feces with the TIANamp Stool DNA Kit, was used as the template for PCR amplification. A PCR master mix was prepared based on a 10 μl final reaction volume containing the Universal SYBR Green mix, extracted DNA, and primer pairs of 16S rRNA gene. Sequences of the primers were as follows: forward primer (515F): 5′-GTGCCAGCMGCCGCGGTAA-3′, reverse primer (805R): 5′-GACTACCAGGGTATCTAATCC-3′. The following cycling conditions were used: 95°C for 30 s and then 42 cycles of 95°C for 10 s, 52.5°C for 20 s, and 72°C for 13 s followed by a melt curve. Three no template controls (NTC) were tested in this experiment, comparatively (Wang et al., 2022).

### GC–MS/MS conditions for CN and SCN quantification

CN in the form of alkylated derivative PFB-CN and SCN in the form of alkylated derivative PFB-SCN were analyzed with a 7890B Gas Chromatograph system combined with a 7000D Triple Quadrupole Mass Spectrometer (Agilent Technologies, Santa Clara, CA, United States). The method was modified according to the previous study and was validated for linearity, accuracy, and precision (Bhandari et al., 2012). The samples were injected at a volume of 1 μl at 2:1 split ratio with an initial temperature of 60°C. The GC oven was equipped with HP-5 ms fused silica capillary column (30 m × 250 μm × 0.25 μm film) coated with 5% diphenyl 95% dimethyl poly siloxane as a stationary phase. The carrier gas used was helium, with a constant flow of 1 ml/min. The temperature gradient of chromatography was programmed as follows: 60°C increased to 130°C at 40°C/min and further increased to a final value of 270°C at 60°C/min (2 min hold). Alkyl derivatives were identified by GC–MS/MS in electron ionization (EI). The optimization of detection was performed by examining of the above ionization types as candidates. MS/MS transitions for each ionization technique were studied with Agilent MassHunter tools (Agilent Technologies, Santa Clara, CA, United States). Fragmentation products were investigated in a full range of collision energies. In the validated method, the detection was performed by multiple reaction monitoring mode (MRM). The temperatures of the transfer line and the ion source were 250°C. Argon was utilized as the collision gas.

### *Ex vivo* incubation of amygdalin and CN

After the determination of concentration, the total protein solution extracted above (called intestinal microbial enzyme, IME) was used for the cyanogenic activity assay (*n* = 4). Varying concentrations of Amy (21.86, 43.73, 109.31, 218.63, 437.25, and 874.51 μM) were mixed into an incubation solution with a final concentration of 0.5 mg/ml IME diluted with 50 mM PBS buffer (PH = 7.4) and incubated at 37°C for 10 min (Hutzler and Tracy, 2002). For the IME of PGF group, 218.63 μM of Amy (approximately Km of Amy in IME of the control group) was mixed for incubation with the same condition, in which other components remained consistent (*n* = 6).

To characterize the ability of intestinal microbiota to convert CN to SCN, we designed another incubation solution (*n* = 6), composed of IME (0.5 mg/ml), 50 mM PBS (pH 7.4), sodium thiosulfate (as the sulfur donor, 6 mM), and CN solution (60 μM, maximum blood exposure concentration in CN toxicokinetics), with the condition of 37°C for 60 min (Adams et al., 2002).

The total volume of the incubation solution was 100 μl and all the incubation solutions above were maintained at 37°C for 3 min before adding substrates. The reaction was initiated by the presence of substrate and stopped by immediately adding 400 μl of ethyl acetate containing IS (290 μM). A derivatization step was as follows: an 800 μl of TBAS (10 mM, in a saturated solution of sodium tetraborate decahydrate, pH 9.5) and a 500 μl of PFB-Br (20 mM, in ethyl acetate) were added to above incubation solution (Bhandari et al., 2012). After a constant temperature shake (60°C, 300 rpm) for 60 min, the solution was centrifuged for 5 min (room temperature) at 10,000 rpm (9,391 ×*g*) to separate the organic and aqueous layers. Finally, the organic layer (200 μl) was collected into an Eppendorf vial with 50 mg anhydrous magnesium sulfate, vortex-mixed, and centrifuged (10,000 rpm for 2 min at room temperature). The prepared extract was placed in chromatographic vials and subsequently analyzed with the gas chromatography-tandem mass spectrometry (GC–MS/MS) system.

### Plasma CN and SCN analysis

Rats (*n* = 6) were administered orally with sub-lethal dose [440 mg/kg (50% LD_50_)]. Blood was drawn before oral administration for the baseline, called as “zero” time point. A 500 μl sample of orbital venous blood was also drawn at 0.5, 1, 1.5, 2, 3, 4, 5, 6, 8, 10, 12, 24, 48, and 72 h post-administration orally. These blood samples were placed in heparinized tubes to prevent coagulation. Plasma samples were separated immediately by centrifuged at 3000 ×*g* for 10 min at 4°C and stored at −80°C until analysis.

### Determination of CN and SCN In feces or cecum contents

Blank fecal samples were freshly collected a day before administration from all rats (*n* = 6). Freshly voided fecal samples were also collected at 6, 12, 24, 48, and 72 h after oral administration of Amy. Cecum contents of the control group were collected immediately for the later assay. All fecal samples or cecum contents were placed in sterile lyophilized tubes and stored at −80°C until analysis.

### β-Glucosidase and rhodanese assay of intestine and liver

To investigate the changes in host metabolic ability to Amy and CN, β-glucosidase and rhodanese of jejunum, ileum, and liver were analyzed (*n* = 6). After the last time point of blood samples, all rats were anesthetized with chloral hydrate (300 mg/kg). The abdominal cavity was opened and liver perfusion was performed with ice-cold saline until it took on an earthy color. And then, the jejunum and ileum were removed and the intestine lining was carefully flushed with ice-cold saline. Finally, the liver and small intestine were transferred to centrifuge tubes and stored at −80°C until analysis. β-glucosidase and rhodanese of jejunum, ileum, and liver were measured using β-glucosidase Assay Kit and Rat Rhodanese ELISA Kit, respectively.

### Intestinal microbiota analysis By 16S rRNA gene sequencing

Total genomic DNA from feces stored at −80°C was extracted using the HiPure Stool DNA Kits (Magen, Guangzhou, China) according to the manufacturer’s protocols (*n* = 6). The 16S rDNA V3-V4 region of the ribosomal RNA gene was amplified by PCR (94°C for 2 min, followed by 30 cycles at 98°C for 10 s, 62°C for 30 s, and 68°C for 30 s, and a final extension at 68°C for 5 min) using primers 341F: CCTACGGGNGGCWGCAG; 806R: GGACTACHVGGGTATCTAAT. PCR reactions were performed in a triplicate 50 μl mixture containing 5 μl of 10 × KOD Buffer, 5 μl of 2 mM dNTPs, 3 μl of 25 mM MgSO4, 1.5 μl of each primer (10 μM), 1 μl of KOD Polymerase, and 100 ng of template DNA. Amplicons (466 bp) were extracted from 2% agarose gels and purified using the AxyPrep DNA Gel Extraction Kit (Axygen Biosciences, Union City, CA, United States) according to the manufacturer’s instructions and quantified using ABI Step One Plus Real-Time PCR System (Life Technologies, Foster City, United States). Purified amplicons were pooled in equimolar and paired-end sequenced (2 × 250) on an Illumina platform according to the standard protocols, and further filtered with FASTP to get high-quality clean reads. Paired-end clean reads were merged as raw tags using FLSAH (version 1.2.11) with a minimum overlap of 10 bp and mismatch error rates of 2%. Noisy sequences of raw tags were filtered by QIIME (version 1.9.1) pipeline under specific filtering conditions to obtain high-quality clean tags. Clean tags were searched against the reference database (version r20110519)^1^ to perform reference-based chimera checking using the UCHIME algorithm. All chimeric tags were removed and finally obtained effective tags were used for further analysis. The effective tags were clustered into operational taxonomic units (OTUs) of ≥97% similarity using UPARSE (version 9.2.64) pipeline. The tag sequence with the highest abundance was selected as the representative sequence within each cluster. Alpha diversity index (Chao1, Simpson and Pielou) were calculated in QIIME (version 1.9.1), and beta diversity (non-metric multidimensional scaling, NMDS) and unweighted pair-group method with arithmeticmeans (UPGMA) was calculated using the vegan package in R-package.

### Metagenomic sequencing

Metagenomic sequencing was performed with qualified genomic DNA extracted from fecal samples (*n* = 3). The genomic DNA was firstly fragmented by sonication to a size of 350 bp, and then end-repaired, A-tailed, and adaptor-ligated using NEBNext® MLtra™ DNA Library Prep Kit for Illumina (NEB, United States) according to the preparation protocol. DNA fragments with a length of 300–400 bp were enriched by PCR. At last, PCR products were purified using the AMPure XP system (Beckman Coulter, Brea, CA, United States) and libraries were analyzed for size distribution by 2100 Bioanalyzer (Agilent, Santa Clara, CA, United States) and quantified using real time PCR. Genome sequencing was performed on the Illumina Novaseq 6000 sequencer using the pair-end technology (PE 150). Raw data from the Illumina platform were filtered using FASTP (version 0.18.0) with general standards. Clean reads of each sample were assembled individually using MEGAHIT (version 1.1.2) stepping over a k-mer range of 21–99 to generate sample-derived assembly. Genes were predicted based on the final assembly contigs (>500 bp) using MetaGeneMark (version 3.38). The predicted genes ≥300 bp in length from all samples were pooled and combined based on ≥95% identity and 90% reads coverage using CD-HIT (version 4.6) to reduce the number of redundant genes for the downstream assembly step. The reads were re-aligned to predicted genes using Bowtie (version 2.2.5) to count reads numbers. The final gene catalogue was obtained from non-redundant genes with gene reads count >2. The unigenes were annotated using DIAMOND (version 0.9.24) by aligning with the deposited ones in diverse protein databases including the National Center for Biotechnology Information (NCBI) non-redundant protein (Nr) database, Kyoto Encyclopedia of Genes and Genomes (KEGG), evolutionary genealogy of genes: Non-supervised Orthologous Groups (eggNOG). Clean reads were used to generate the taxonomic profile using Kaiju (version 1.6.3).

### Data processing and statistical analysis

Pharmacokinetic parameters of CN and SCN in PGF group were estimated by noncompartmental analysis using Phoenix WinNonlin software version 8.3.1.5014 (Pharsight, Mountain View, CA, United States). All Data were expressed as mean ± standard deviation (SD). The data for BCA, qPCR, incubation experiment and toxicokinetics experiment were analyzed by unpaired t-test using SPSS version 26.0 (IBM, Armonk, NY, United States), and a *p* value less than 0.05 was considered statistically significant. Statistical significance for alpha and beta-diversity was determined by Welch’s t-test with R-package and GraphPad Prism version 8.0 (GraphPad Software, La Jolla, CA, United States). The correlation analysis was conducted with Pearson correlation coefficients, and the R-package (Corrplot, ggcorplot, and pheatmap) was used for the correlation matrix visualization. The relative abundance of sulfurtransferase superfamily, rhodanese, β-glucosidase, *Escherichia coli* and Nitrogen metabolism pathway were compared by unpaired t-test using SPSS version 26.0 (IBM, Armonk, NY, United States).

## Results

### Animal conditions during feeding and experiment

**Figure 2:**
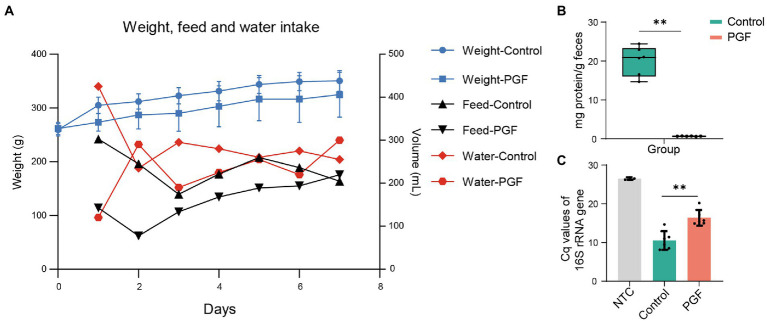
**(A)** Weight, feed and water intake in the control and PGF groups during antibiotics-treatment. The body weight was shown as mean ± SD (*n* = 6). Data of water and feed intake was shown as the sum of consumption in the respective group. **(B)** Total enzyme content in feces (*n* = 6); **(C)** Relative abundance of total DNA of two groups evaluating by the 16S rRNA gene Cq values (*n* = 6). Data of samples was shown as black dots, and the error bars denoted SD, *p < 0.05 and **p < 0.01.

To observe the living state of rats, the feed intake, water intake, and body weight were recorded during antibiotic-treatment (Figure 2A). The body weight of rats in the control group was higher than that in the PGF group and both went up over time. The water and feed intake showed a large difference between the two groups on the first day, and the difference narrowed later. It might be that the rats of the PGF group initially developed a taste resistance to the antibiotic cocktail, and then gradually adapted.

The rats in the control group developed acute CN poisoning symptoms after administration of Amy for 1.5–2 h, such as respiratory distress, generalized convulsions, and weakness. However, there was no apparent toxic reaction in the PGF rats during the whole experiment, and keep alive until the end of the experiment.

### Changes in total abundance of intestinal microbiota with antibiotic-treatment

There was a significant reduction in IME concentration after treatment by broad-spectrum antibiotics for 7 days (Figure 2B). QPCR for feces showed that the quantification cycle (Cq) of the PGF group for the 16S rRNA gene increased by 55.65% compared with that of the control group (Figure 2C), which was fundamentally consistent with our previous study (Wang et al., 2022).

### Method validation

**Figure 3:**
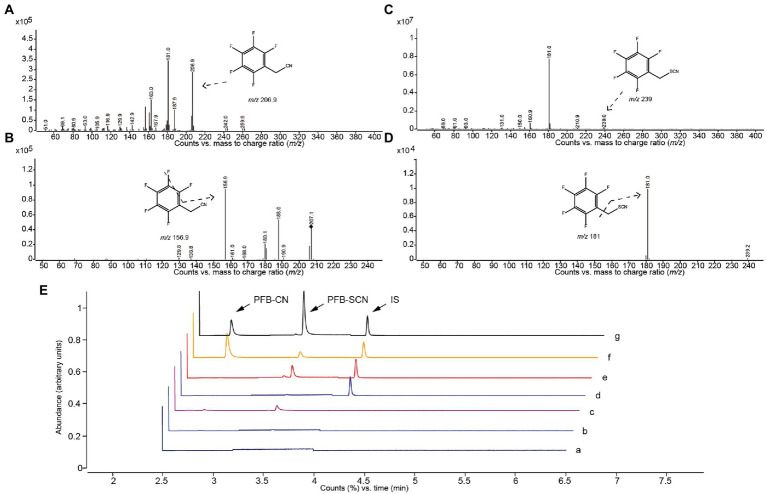
Mass spectrogram and chromatographs of alkylated CN and SCN. Abundance of **(A)** parent ions and **(B)** daughter ions of PFB-CN; Abundance of **(C)** parent ions and **(D)** daughter ions of PFB-SCN. GC–MS/MS chromatographs **(E)** of PFB-CN in (a) solvent, (b) non-spiked intestinal microbial enzyme solution, (c) non-spiked plasma, (d) is-spiked intestinal microbial enzyme solution, (e) is-spiked plasma, (f) intestinal microbial incubation sample, and (g) plasma sample.

Characteristic ion fragments of PFB-CN and PFB-SCN were shown in Figures 3A–D; Table 1. The representative MRM chromatograms of analytes and IS spiked in matrix (plasma and IME) were shown in Figure 3E.

**Table 1:** Main parameters of alkylated CN and SCN.

Analytes	Alkylated derivative	Retention time (min)	Parent ions (m/z)	Daughter ions (m/z)
CN	PFB-CN	2.88	206.9	156.9*, 188
SCN	PFB-SCN	3.95	238.9	181*, 160.9

*Ions for quantification.

The derivatized CN and SCN both showed a good linear relationship. Calibration plots were constructed in the CN concentration ranges of 2.34–600.00 μM for the IME and 1.56–100.00 μM for the plasma and in the SCN concentration ranges of 0.58–600.00 μM for the IME and 6.25–800.00 μM for the plasma. The results of accuracy and precisions of the method were summarized in Table 2.

**Table 2:** Accuracy of CN and SCN in intestinal microbial enzyme solution and plasma (*n* = 6).

Analytes	Matrix					
	Intestinal microbial enzyme solution			Plasma		
	Concentration (μM)	Accuracy (%) (mean ± SD)	RSD (%)	Concentration (μM)	Accuracy (%) (mean ± SD)	RSD (%)
CN	5	90.20 ± 11.15	12.36	2	106.91 ± 5.78	5.40
	10	94.28 ± 9.9	10.49	10	109.55 ± 5.32	4.85
	50	112.59 ± 3.04	2.70	75	96.83 ± 14.21	14.67
	500	115.39 ± 3.05	2.65			
SCN	5	104.80 ± 7.79	7.43	20	86.48 ± 4.39	5.07
	10	98.40 ± 2.22	2.26	100	101.01 ± 2.66	2.64
	50	100.59 ± 1.37	1.36	600	104.23 ± 4.87	4.67
	500	95.43 ± 2.73	2.86			

### Enzymatic hydrolysis rate of Amy and CN in IME

**Figure 4:**
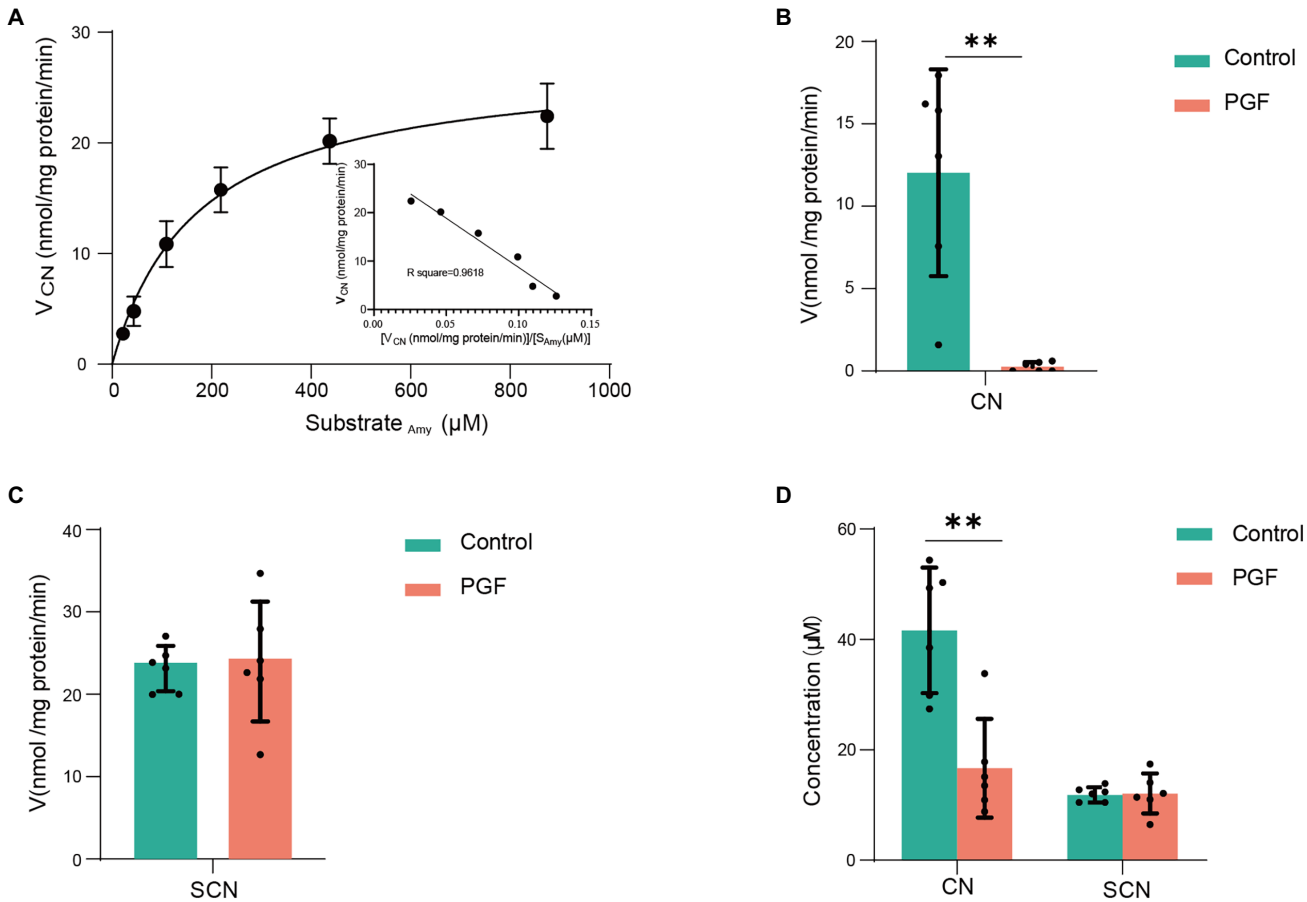
Enzyme kinetic characteristics of intestinal microbiota from the control and PGF groups. **(A)** Double Michaelis–Menten equation (*n* = 4) for normal intestinal microbial enzyme kinetics, black dots denoted mean, and error bars denoted SD; **(B)** Generation rate of CN from amygdalin in the intestinal microbial enzyme system (*n* = 6); **(C)** Generation rate of SCN from CN in the intestinal microbial enzyme system (*n* = 6); **(D)** Final concentration of CN and SCN after incubating with 60 μM of CN in the intestinal microbial enzymes (*n* = 6). Data was shown as mean ± SD, black dots denoted samples, and the error bars denoted SD, *p < 0.05 and **p < 0.01.

Enzymatic kinetics of Amy in the IME of the control group, represented in terms of the generation rate of CN, was shown in Figure 4A. The best fit of data shown was obtained using the double Michaelis–Menten equation for enzyme kinetics. The Vmax and Km were, respectively, 27.56 ± 4.13 nmol/(mg protein)/min and 173.78 ± 39.16 μM. In contrast to the control group, the release rate of CN (incubated with Amy, 218.63 μM) was significantly inhibited in the PGF group, and the generation rate of SCN (CN as substrate) was also significantly inhibited (Figures 4B,C). Figure 4D showed no significant difference in the generation of SCN between the two groups, while a stronger metabolic capacity of CN was shown in the intestinal microbiota of the PGF group.

### Toxicokinetics of amygdalin

**Figure 5:**
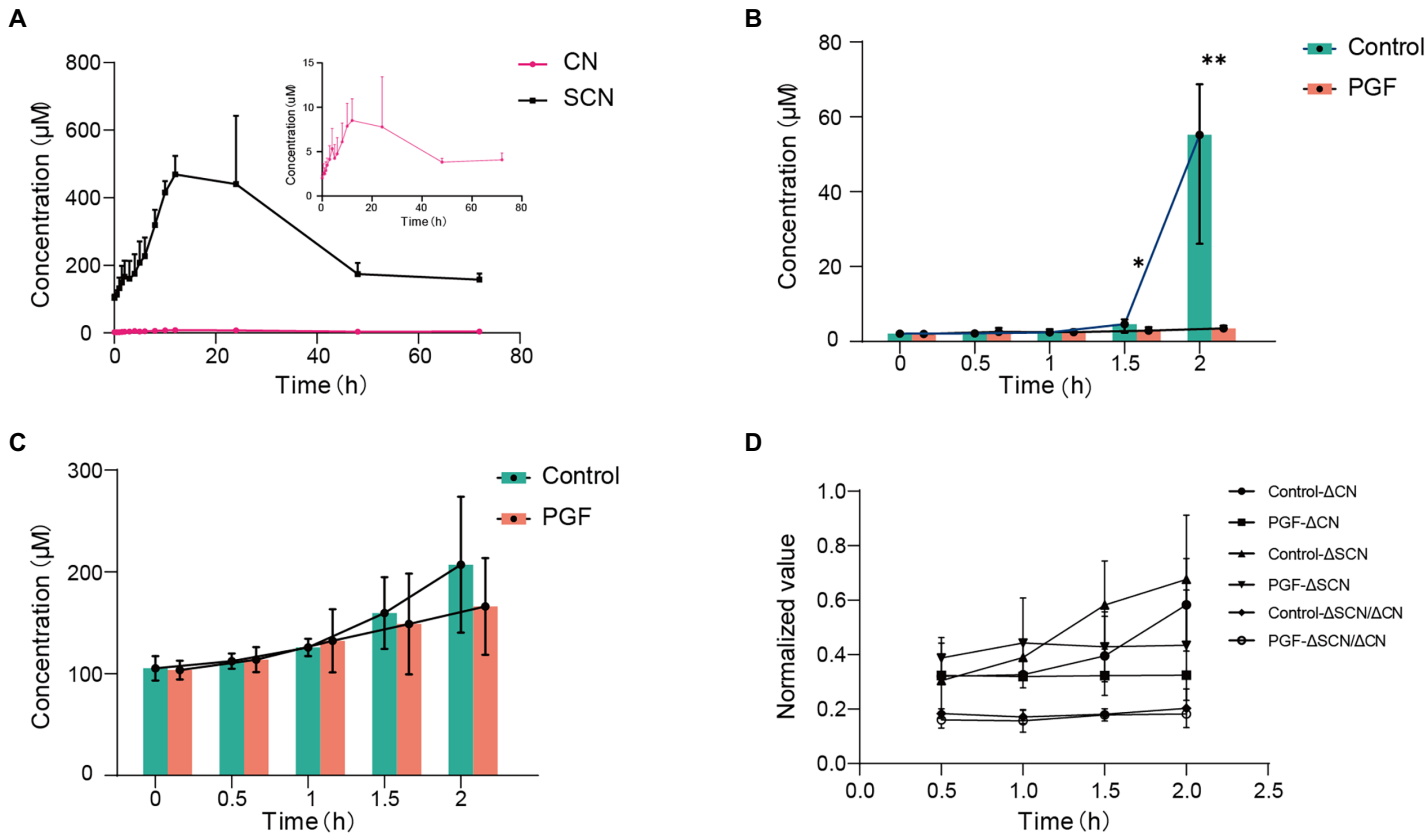
Mean plasma concentration-time profiles of CN and SCN after oral administration of amygdalin (*n* = 6). **(A)** Overview of mean plasma concentration profiles over time for CN and SCN in the PGF group; Mean plasma concentration profiles for **(B)** CN and **(C)** SCN at 0, 0.5, 1, 1.5, 2 h after oral administration of amygdalin; **(D)** Increase of CN and SCN in plasma for every 0.5 h within 2 h, and the relative speed of increase was represented by their ratio. Δ: increase in plasma concentration relative to the previous sampling point. *p < 0.05 and **p < 0.01.

The plasma concentration-time curve of CN and SCN and corresponding pharmacokinetics in PGF rats after the oral administration of Amy was displayed in Figure 5A; Table 3. In comparison with PGF rats, a sharp increase (*p* < 0.05) was observed in the plasma concentration of CN in the control group after 1.5 h and 2 h of the administration of Amy (Figure 5B), causing fatal toxicity. However, there was no significant difference in the plasma concentration of SCN (Figure 5C). The rate of increase of CN and SCN in plasma within 2 h after administration of Amy was shown in Figure 5D. The results suggested that the generation rate of SCN seem to be steady unless an extremely increase of CN.

**Table 3:** Pharmacokinetics parameters of CN and SCN after oral administration of amygdalin for PGF group (mean ± SD, *n* = 6).

Analytes	C_max_ (μM)	T_max_ (h)	AUC_(0-t)_ (h•μM)	AUC_(0-∞)_ (h•μM)	T_1/2_ (h)	V_d_ (mL•kg^−1^)	CL (mL•h^−1^ •kg^−1^)
CN	10.1 ± 5.1	13.7 ± 5.1	396.0 ± 101.0	894.0 ± 275.0	82.3 ± 40.2	2192307.7 ± 757692.3	20076.9 ± 4692.3
SCN	514.0 ± 158.0	13.7 ± 5.1	20000.0 ± 3810.0	28000.0 ± 2840.0	34.6 ± 9.6	13637.9 ± 3741.4	274.1 ± 30.0

### CN and SCN in feces or cecum contents

**Figure 6:**
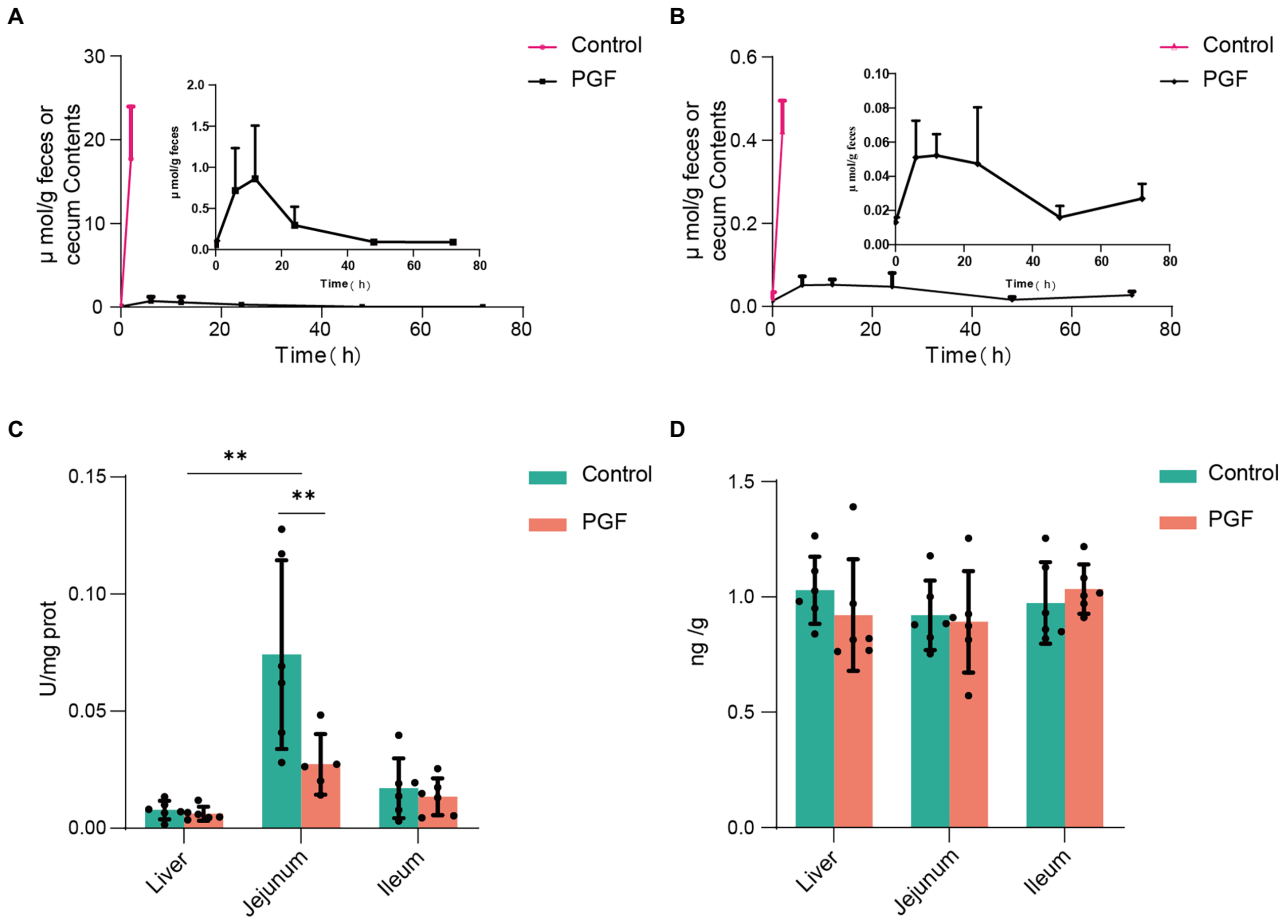
Fecal excretion of CN and SCN and the host CN and SCN metabolic enzyme activity (*n* = 6). **(A)** CN and **(B)** SCN content in the caecum (for the control group) or feces (for the PGF group) over time; **(C)** Activity of β-glucosidase; **(D)** Expression of rhodanese in liver, jejunum, and ileum. *p < 0.05 and **p < 0.01.

Figures 6A,B showed the levels of CN and SCN in the cecum contents from the control group or feces from the PGF group after oral administration of Amy. The result illustrated that the fecal excretion of CN and SCN in rats may be ignored. However, CN and SCN in the cecum contents of the control group were detected at a quite high level. The reasonable conjecture was made according to the above results, that the CN and SCN in the cecum were most likely produced by the metabolism of intestinal microbiota in the control group.

### Alteration of β-glucosidase and rhodanese in small intestine and liver with antibiotic-treatment

Figures 6C,D showed the activity of β-glucosidase and expression of rhodanese in the liver, jejunum, and ileum tissue from the control and PGF group. It was apparent that the activity of β-glucosidase in the jejunum of rats was higher than that of the ileal segment and liver, and the antibiotic treatment significantly inhibited the activity of β-glucosidase in the jejunum of rats and not the ileum or liver, resulting in the comparable activity of β-glucosidase in jejunal and ileal in the PGF group. The expression of rhodanese was essentially the same in the main metabolic organs of the rats, including the liver, ileum, and jejunum tissue, and was unaffected by antibiotic treatment.

### Alteration of intestinal microbiota with antibiotic-treatment

**Figure 7:**
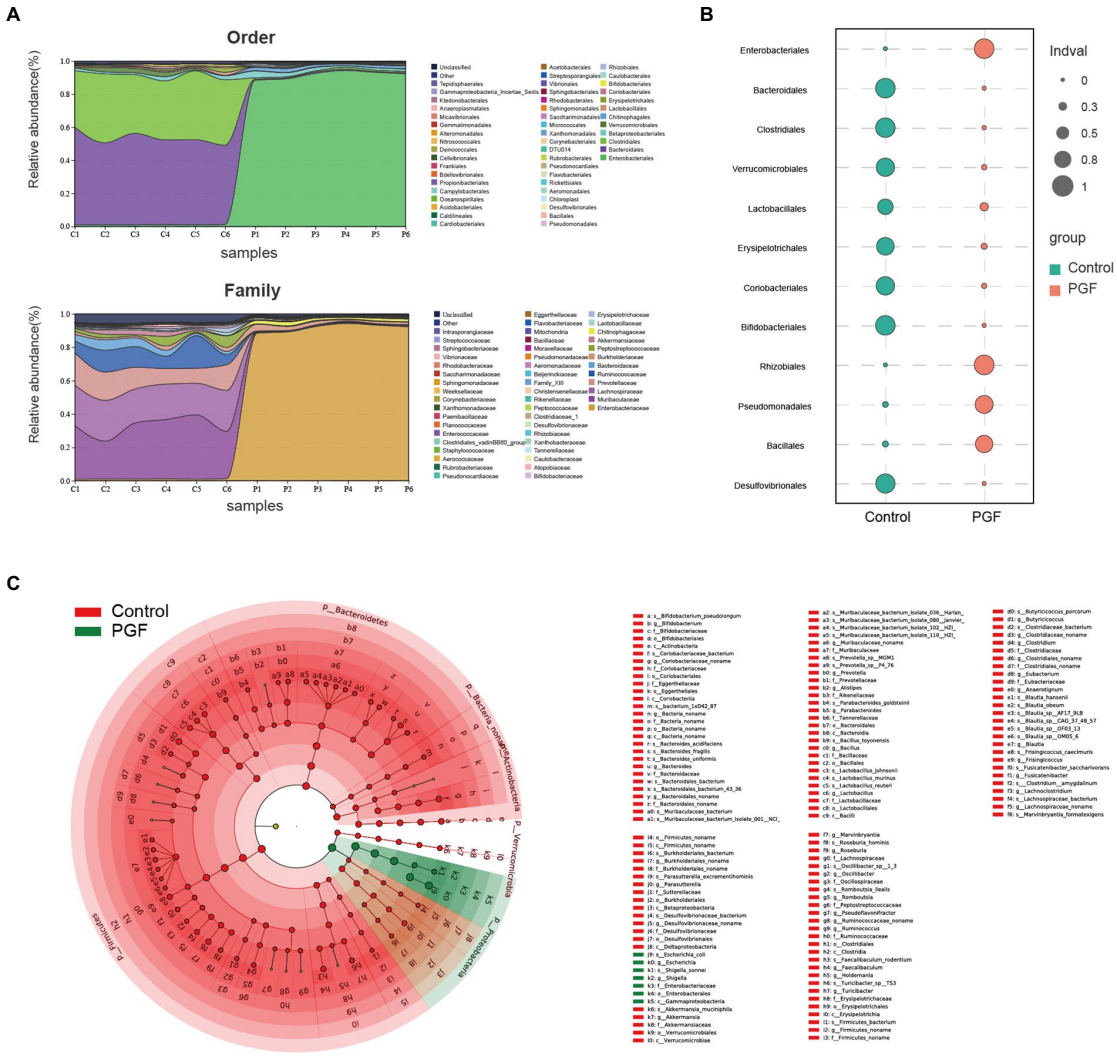
Microbiota characteristics of the feces from the control and PGF groups. **(A)** Distribution of bacterial orders and families (*n* = 6); **(B)** Indicator taxa at order level based on the specie abundance and frequency of occurrence in two groups (*n* = 6); **(C)** Different species based on the Linear discriminant analysis Effect Size (LEfSe) analysis, linear discriminant analysis (LDA) score > 2 and *p <* 0.05 (*n* = 3).

16S rRNA gene sequencing of the feces from the control and PGF groups was performed to investigate the effects of antibiotic treatment. Figure 7A showed the microbial distribution in the taxa at order and family levels. Indicator taxa at order level of two groups were shown in Figure 7B. Differences between the two groups were analyzed by Linear discriminant analysis Effect Size (LEfse), and the main taxa specific to each group [Linear discriminant analysis (LDA) score > 2] were identified by metagenomic sequencing (Figure 7C).

**Figure 8:**
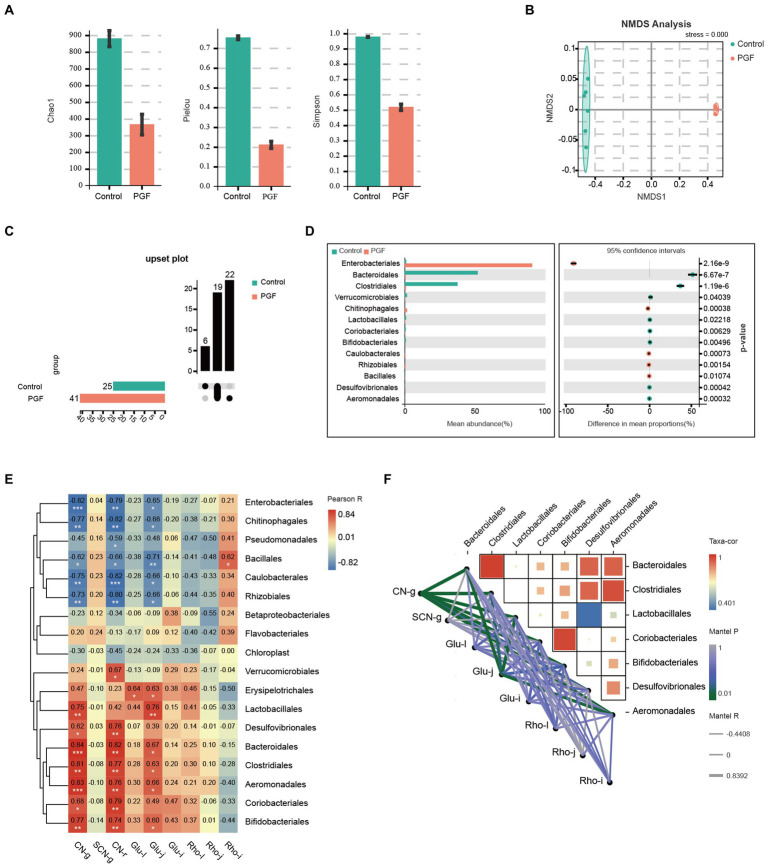
Intestinal microbiota composition difference of the control and PGF groups (*n* = 6). **(A)** Chao1, Pielou, and Simpson indexes (*p <* 0.01); **(B)** Non-metric multidimensional scaling (NMDS) plot based on Bray-Curtis distances comparing microbiota composition between the control and PGF group, stress = 0.000, *p <* 0.01; **(C)** Upset plot based on bacterial orders; **(D)** Different taxa at order level, *p <* 0.05; **(E)** Correlation heatmap between the detected indexes and bacterial orders based on Pearson correlation coefficient, *p < 0.05, **p < 0.01, and ***p < 0.001; **(F)** Correlation heatmap between bacterial orders and bacterial orders determined based on correlation coefficient and *p* value, and correlation network between bacterial orders and detected indexes based on Pearson correlation coefficient and *p* value. CN-g and SCN-g: concentrations of CN and SCN in the incubation experiment; glu-l, glu-j, and glu-i: β-glucosidase activity of liver, jejunum, and ileum; rho-l, rho-j, and rho-i: rhodanese expression of liver, jejunum, and ileum; Taxa-cor: correlation coefficient between bacterial orders based on Pearson correlation analysis; Mantel P: *p* value of Mantel test between taxa and the metabolic indicators based on Pearson correlation analysis; Mantel R: correlation coefficient of Mantel test between taxa and the metabolic indicators based on Pearson correlation analysis.

Intestinal microbiota composition of the PFG group was characterized by a decline in α diversity indexes comprising Chao1, Pielou, and Simpson indexes compared with the control group (Figure 8A), indicating a significant reduction in the abundance and uniformity of intestinal microbiota in the PGF group. We further investigated the different species at order level between the control and PGF groups. NMDS analysis showed that the β diversity of bacterial communities in two groups were obviously separated (Figure 8B). The amounts of different bacterial taxa at order level were shown in Figure 8C, and 13 of these orders turned out to be significantly different in the relative abundance (Figure 8D). To further investigate the main bacterial orders causing the differences in metabolic indicators (including the generation of CN and SCN *ex vivo* and the relative metabolic enzymes activity and expression in the liver, jejunum, and ileum of rats) between the two groups, Pearson correlation analysis was performed based on the abundance of bacterial orders (Figures 8E,F). The results demonstrated that there were 7 orders (*Aeromonadales*, *Bacteroidales*, *Bifidobacteriales*, *Clostridiales*, *Coriobacteriales*, *Desulfovibrionales*, and *Lactobacillales*) shown potential correlation with cyanogenesis, and 6 orders (*Aeromonadales*, *Bacteroidales*, *Bifidobacteriales*, *Clostridiales*, *Erysipelotrichales*, and *Lactobacillales*) might provide explanations for the change in β-glucosidase activity of jejunum (*p* < 0.05).

### Gene function analysis based on metagenomic sequencing

**Figure 9:**
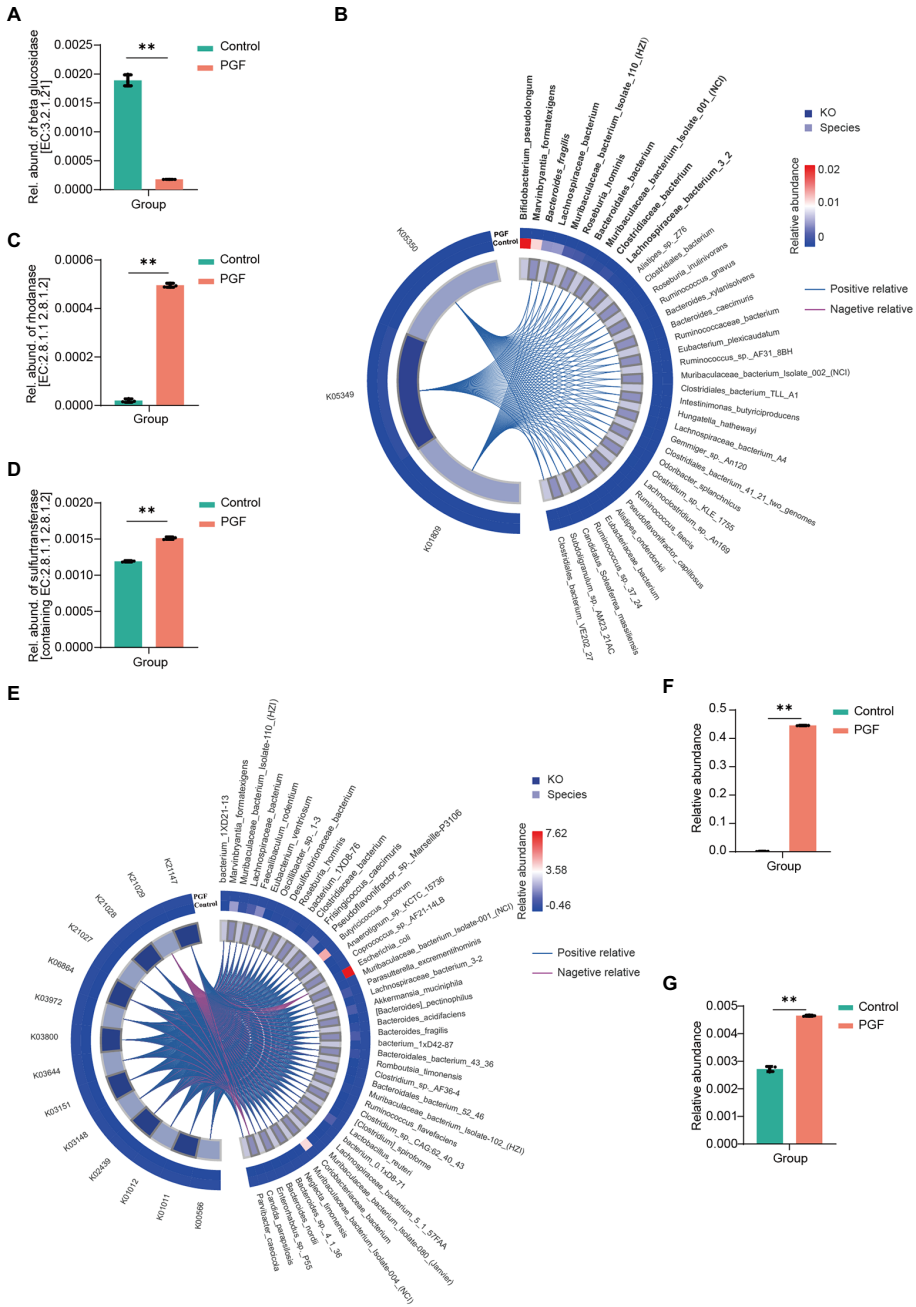
Identified species and annotated gene based on the metagenomic gene sequencing (n = 3). **(A)** Relative abundance of β-glucosidase (EC:3.2.1.21); **(B)** Correlation between β-glucosidase-related KEGG Orthologs (KO) and bacterial species (Top10 species were shown in bold, and italics were reported in previous studies), based on the determined 7 orders relative to cyanogenesis of amygdalin by Pearson correlation analysis; **(C)** Relative abundance of genes encoding putative rhodanese (EC:2.8.1.1 2.8.1.2), *p <* 0.01; **(D)** Relative abundance of genes encoding all the sulfurtransferases (including EC:2.8.1.1 2.8.1.2), *p <* 0.01; **(E)** Correlation between sulfurtransferase-related KO and the bacterial species identified by metagenomic sequencing; **(F)** Relative abundance of *Escherichia coli*; **(G)** Relative abundance of Nitrogen metabolism pathway *via* KEGG enrichment.

To fine-tune the identification of key species causing metabolic differences in CN and SCN and the expression of related functional genes, metagenomic sequencing was conducted. β-glucosidase (EC:3.2.1.21) was known as a critical enzyme in the cyanogenic metabolism of Amy. We accordingly explored the genes encoding the putative β-glucosidase based on the gene annotation prediction with KEGG. It was found that the relative abundance of genes encoding the putative β-glucosidase in the control group was significantly higher than in the PGF group (Figure 9A). We further mapped the genes encoding the putative β-glucosidase to the species among the 7 orders concerned with cyanogenesis to find the main species (Supplementary Table S1). The relative abundance of the species was shown in Figure 9B, among which *Bacteroides fragilis* has been reported to be more potential to catalyze the cyanogenesis of Amy (Cressey and Reeve, 2019). Furthermore, we investigated the potential detoxification enzyme of CN. The results showed that the relative abundance of genes encoding the putative rhodanese/3-mercaptopyruvate sulfurtransferase (EC:2.8.1.1/2.8.1.2) in the PGF group was significantly higher than those in the control group, which cannot fully explain the generation of SCN *ex vivo* (Figure 9C). Then the relative abundance of sulfurtransferase superfamily was further analyzed (Figure 9D). The results suggested that there may be other enzymes in the sulfurtransferase superfamily that can convert CN to SCN. The species corresponding to the genes encoding putative sulfurtransferases were shown in Figure 9E; Supplementary Table S2.

Additionally, we noticed that CN in the PGF group exhibited some additional consumption except conversion to SCN in our incubation experiment *ex vivo* (Figure 4F). Different from SCN pathway, this part of CN was likely to be assimilated by some amino acids or peptides, or involved in the transformation of nitrogen metabolism into ammonia. Another detoxification enzyme of CN called L-3-cyanoalanine synthase (EC:4.4.1.9), came to our mind, catalyzing the conversion of L-3-cyanoalanine cyanalanine and H2S from L-cysteine and CN, and was widely found in higher plants and some bacteria, such as *E. coli* (Dunnill and Fowden, 1965). Interestingly, *E. coli* in the PGF group was significantly enriched in comparison with the control group, and the relative abundance of nitrogen metabolism pathway annotated in the PGF group rose significantly (Figures 9F,G), which could be another explanation for the higher level of CN detoxification metabolism in the PGF group.

## Discussion

The metabolites of Amy were reported increasingly in recent years, including prunasin, mandelonitrile benzaldehyde, Amy amide, prunasin amide, and so on (Qin et al., 2021). It has been proved that CN metabolism pathway is most closely associated with toxicity of Amy (Cmorej et al., 2022). In this study, the rats in the control group showed typical symptoms of CN poisoning after oral administration of Amy, including respiratory distress, generalized convulsions, and weakness, and was accompanied by a significant increase in the plasma concentration of CN (Ortega and Creek, 1978; Parker-Cote et al., 2018). Instead, the rats in the PGF group showed no sign of poisoning during the experiment.

It was reported that the mean median lethal dose (LD50) of Amy in rats was found to be approximately 880 mg/kg body weight by oral administration (Adewusi and Oke, 1985). In our study, oral administration of a sub-lethal dose (50% of LD50) of Amy was not enough to kill all rats in the control group theoretically. The difference may not be only attributed to the drug itself (purity or extraction methods) but also related to other factors that affect detoxification metabolism, such as intestinal microbiota. The incubation experiment showed that intestinal microbiota had great potential in cyanogenesis of Amy. It is well known that intestinal microbiota is influenced by various external environments, such as feeding environment, diet, and age (Hopkins et al., 2001; Mccracken et al., 2001; Favier et al., 2002), and the lethal dose of Amy could be impacted. In our study, β-glucosidase activity in the small intestine of rats of the control and PGF groups showed a significant difference, suggesting that the composition of intestinal microbiota may affect the β-glucosidase of the small intestine, which may be another reason for the different observations in the LD50 of Amy. In addition to the cyanogenesis pathway on the LD50 of Amy, the detoxification pathway of CN should also be considered, such as SCN metabolism pathway. SCN metabolism pathway not only includes the role of the host but also the impact of intestinal microbiota, which was more prone to individual differences. Unfortunately, we failed to evaluate its detoxification potential *in vivo* due to acute toxicity in rats resulting in death.

Some intestinal bacteria (*Enterobacter aerogenes*, *Streptococcus fecalis*, *Clostridium perfringens*, etc.) from human feces were found to be capable of releasing CN from Amy, with *B. fragilis* more efficiently, but *E. coli* did not liberate CN (Cressey and Reeve, 2019). In this study, apart from *B. fragilis*, additional species associated with cyanogenesis of Amy were identified in an unculture manner, which further enriched the bacteria spectrum with the putative cyanogenesis function. SCN metabolism pathway, considered as a natural barrier to the toxicity of CN (host and intestinal microbiota), was overlooked. In the toxicokinetics analysis, CN and SCN levels in the PGF group increased at a steady rate within 2 h after oral administration of Amy. In contrast, plasma CN and SCN levels increased rapidly in the control group. The results indicated a more effective barrier against Amy toxicity in the PGF group, including thiocyanate conversion and the inhibition of cyanogenesis process, which might be related to the enrichment of *E. coli* in the intestine. The ratio of plasma SCN to CN increment of every 0.5 h was compared, the value in the PGF group was lower than that of the control group within 1 h of oral administration of Amy, and then gradually outperformed the control group, which meant intestinal microbiota performed a pre-barrier of CN toxicity before a sharp increase in plasma CN concentration (Figure 5D). Previous studies mostly attributed SCN detoxification pathway to the host, ignoring the role of intestinal microbiota. To investigate whether intestinal microbiota was directly involved in the metabolism of Amy, CN and SCN levels in the feces or cecum contents were detected. Before this, it is critical to determine whether these metabolites will be excreted in the feces. The result of the excretion study confirmed that the SCN was hardly excreted in the feces, which was consistent with previous studies (Himwich and Saunders, 1948; Kirman et al., 2018). The incubation experiment of CN *ex vivo* showed that about 1/3 of the CN was converted to SCN. And the contents of CN and SCN in the cecum contents of the control group were, respectively, 19.89 ± 2.73 μmol/(g caecum contents) and 0.44 ± 0.07 μmol/(g caecum contents) at 2 h after the oral administration of Amy, suggesting that SCN could be generated in the intestine before absorption into the blood, which provided direct evidence for the involvement of intestinal microbiota in Amy detoxification. It was worth mentioning that the SCN in feces was only the portion unabsorbed, not all that was generated.

Other than SCN detoxification pathway, another detoxification pathway of CN was investigated in our study. Previous studies on the cyanoamino acid metabolism pathway of CN were mostly conducted in plants, rarely considering animals and microorganisms, which was associated with the natural CN resistance mechanism of plant cells. In our study, the IME in the control group did not show excessive metabolism of CN except SCN metabolism pathway until the significant enrichment of *E. coli* in the PGF group. In the normal intestinal microbiota of humans or other animals, *E. coli*, classified as a conditional pathogenic bacteria will remain at a low abundance to maintain a state of equilibrium, which means there will be no obvious assimilation of CN. That is, SCN detoxification pathway matters more compared with cyanoamino acid metabolism pathway in humans and other animals with normal intestinal microbiota. Distinguishing the contribution of host and intestinal microbiota to this metabolism pathway and assessing their interaction is still a challenging job and worthy of further investigation.

**Figure 10:**
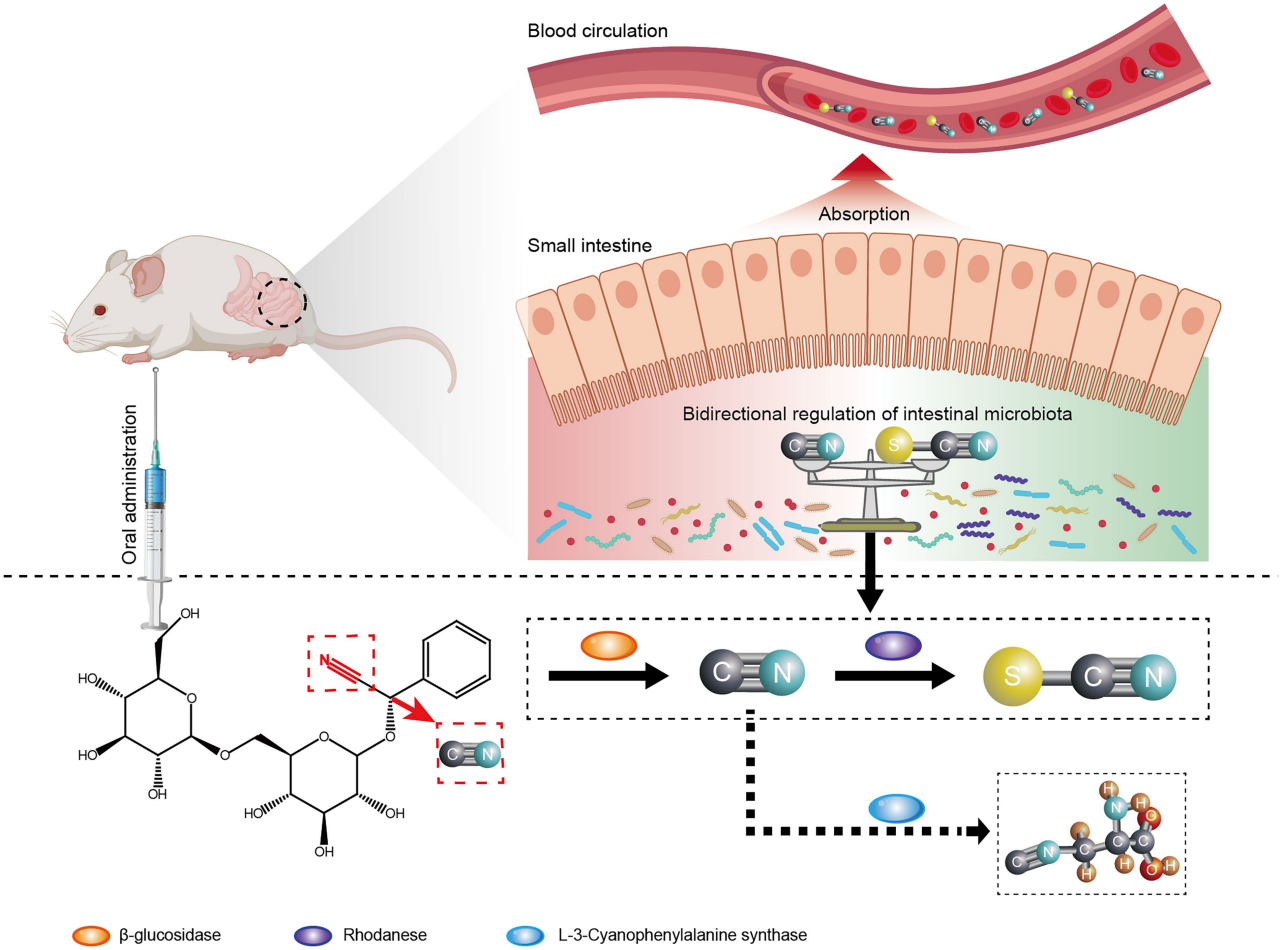
Diagram showing the role of intestinal microbiota on the regulation of toxicity and detoxification mechanisms of amygdalin.

In conclusion, we identified additional cyanogenesis-related species by 16S rRNA gene and metagenomic sequencing, further expanding the cyanogenic bacteria spectrum. SCN metabolism pathway in intestinal microbiota was investigated, and potential involvement of sulfurtransferase superfamily was also discussed. In addition, cyanoamino acid metabolism pathway for CN detoxification may occur in the intestinal microbiota. Therefore, intestinal microbiota might mediate the bidirectional regulation of Amy toxicity and detoxification (Figure 10). When it comes to human beings, with the same or more complex composition and functions, a more complex disposition of drugs is possible, such as metabolic toxicity and detoxification, which may contribute to the uncovering of the complete metabolic process following the oral administration of Amy and other drugs. In addition, we found that intestinal microbiota may also affect metabolic enzyme activity, such as β-glucosidase, in the small intestine and further research will be performed to explore the specific mechanisms involved.

## Data availability statement

The data presented in the study are deposited in NCBI Sequence Read Archive (SRA) with BioProject number: PRJNA886069.

## Ethics statement

The animal study was reviewed and approved by Experimental Animal Ethics Committee of Anhui Medical University.

## Author contributions

SS and CS conceived the idea and designed the study. QW conducted the experiments, analyzed the data, and wrote the manuscript. SY provided technical support, performed data analysis, and revised the manuscript. ShaW, YQ, QX, SheW, and GC participated in the experiments. All authors contributed to the article and approved the submitted version..

## Funding

This study was financially supported by National Natural Science Foundation of China (no. 81703800), Anhui University of Traditional Chinese Medicine Graduate School of Pharmacy Training Fund Project of China (no. 21pyjj06) and the First Affiliated Hospital of Anhui Medical University for the Cultivation Program of Youth Science Foundation of China (no. 2792).

## Conflict of interest

The authors declare that the research was conducted in the absence of any commercial or financial relationships that could be construed as a potential conflict of interest.

## Publisher’s note

All claims expressed in this article are solely those of the authors and do not necessarily represent those of their affiliated organizations, or those of the publisher, the editors and the reviewers. Any product that may be evaluated in this article, or claim that may be made by its manufacturer, is not guaranteed or endorsed by the publisher.

## Footnotes

^1^ http://drive5.com/uchime/uchime_download.html
