# Corrigendum: Impact of intestinal microbiota on metabolic toxicity and potential detoxification of amygdalin

**DOI:** 10.3389/fmicb.2023.1303273

**Published:** 2023-11-01

**Authors:** Qiuyu Wen, Shen Yu, Shanshan Wang, Yan Qin, Quan Xia, Sheng Wang, Guanjun Chen, Chenlin Shen, Shuai Song

**Affiliations:** ^1^School of Pharmacy, The First Affiliated Hospital of Anhui Medical University, Anhui Medical University, Hefei, China; ^2^The Grade 3 Pharmaceutical Chemistry Laboratory of State Administration of Traditional Chinese Medicine, Hefei, China; ^3^Center for Scientific Research of Anhui Medical University, Hefei, China

**Keywords:** amygdalin, toxicokinetic, intestinal microbiota, cyanide, detoxification

In the published article, there was an error in the affiliations. Instead of “^1^Inflammation and Immune Mediated Diseases Laboratory of Anhui Province, Anhui Institute of Innovative Drugs, Institute for Liver Diseases of Anhui Medical University, School of Pharmacy, Anhui Medical University, Hefei, China, ^2^Department of Pharmacy, The First Affiliated Hospital of Anhui Medical University, Hefei, China” it should be “^1^School of Pharmacy, The First Affiliated Hospital of Anhui Medical University, Anhui Medical University, Hefei, China.”

The authors apologize for this error and state that this does not change the scientific conclusions of the article in any way. The original article has been updated.

